# Diagnosis of pneumonia from chest X-ray images using YOLO deep learning

**DOI:** 10.3389/fnbot.2025.1576438

**Published:** 2025-04-28

**Authors:** Yanchun Xie, Binbin Zhu, Yang Jiang, Bin Zhao, Hailong Yu

**Affiliations:** ^1^Department of Orthopaedics, General Hospital of Northern Theater Command, Shenyang, China; ^2^Department of Endocrinology, The Fourth People's Hospital of Shenyang, Shenyang, China; ^3^Faculty of Robot Science and Engineering, Northeastern University, Shenyang, China

**Keywords:** classification detection, pneumonia detection, YOLO model, deep learning, pneumonia diagnosis

## Abstract

Early and accurate diagnosis of pneumonia is crucial to improve cure rates and reduce mortality. Traditional chest X-ray analysis relies on physician experience, which can lead to subjectivity and misdiagnosis. To address this, we propose a novel pneumonia diagnosis method using the Fast-YOLO deep learning network that we introduced. First, we constructed a pneumonia dataset containing five categories and applied image enhancement techniques to increase data diversity and improve the model’s generalization ability. Next, the YOLOv11 network structure was redesigned to accommodate the complex features of pneumonia X-ray images. By integrating the C3k2 module, DCNv2, and DynamicConv, the Fast-YOLO network effectively enhanced feature representation and reduced computational complexity (FPS increased from 53 to 120). Experimental results subsequently show that our method outperforms other commonly used detection models in terms of accuracy, recall, and mAP, offering better real-time detection capability and clinical application potential.

## 1 Introduction

In recent years, with the rapid development of deep learning technologies, the application of artificial intelligence in medical image diagnosis and analysis has made significant progress, particularly in disease prevention and diagnosis. Pneumonia, an acute respiratory infection caused by bacteria, viruses, or other pathogens, is typically diagnosed through chest X-rays. As a widely used and common medical imaging technique, chest X-rays are crucial in the early screening and diagnosis of respiratory diseases such as pneumonia. Pulmonary opacities or inflammatory lesions characterize the typical chest X-ray presentation of pneumonia. Due to the clinical manifestations of pneumonia often resembling those of other pulmonary diseases, coupled with the increasing number of cases and the accumulation of medical imaging data, the workload of radiologists has steadily increased. Traditional manual interpretation is often limited, and the efficiency and accuracy of human diagnoses are challenged. Therefore, developing an automated diagnostic system based on deep learning can assist physicians in efficiently screening imaging data and providing remote medical support in underserved regions, reducing misdiagnosis rates and enhancing the overall quality of healthcare services. YOLO (You Only Look Once), as an efficient and real-time object detection algorithm, has achieved promising applications in various fields. Its simple structure and fast processing speed make it well-suited for handling large-scale medical imaging data, enabling precise localization and classification of lesion areas quickly. In the application of pneumonia diagnosis, the YOLO algorithm can accurately identify pneumonia lesions by analyzing the detailed features in chest X-ray images, assisting physicians in making rapid and accurate diagnoses. Further optimization of the YOLO model’s detection accuracy and robustness in complex environments, coupled with the characteristics of medical imaging, is expected to promote the development of automated pneumonia X-ray image recognition and diagnosis, facilitating the transition of medical image diagnosis from experience-driven to data-driven intelligence. Research on pneumonia diagnosis has been conducted in studies focused on depth image recognition detection, as shown by Rajendra et al. (2025); Garg et al. (2025); Yi et al. (2025); Buriboev et al. (2024); Sharma and Guleria (2024). For example, Dey et al. (2021) proposed an improved VGG19 deep learning architecture for diagnosing chest X-rays, introducing a feature integration scheme that combines deep and handcrafted features for diagnosing lung abnormalities. Kulkarni et al. (2023) explored how to integrate variational quantum circuits into classical neural networks for pneumonia detection from chest X-rays, showing that the hybrid network outperformed classical networks on various performance metrics. Majumder et al. (2024) employed a split learning scheme to address the issue of limited medical data at individual hospitals, training a single model on a server. This study utilizes an asynchronous split learning approach to overcome challenges posed by unreliable network connections, ensuring the learning process can continue even during a network failure. Raghaw et al. (2024) proposed a novel explainable contrast-based extended convolution network (XCCNet) for pediatric pneumonia detection, addressing issues with low radiation intensity and traditional image processing methods that are time-consuming and fail to capture prominent features. By integrating feature visualization and explainability methods, this approach directly aligns with the regions of interest on the X-ray images that indicate the presence of pneumonia or normality. Extensive evaluations on four datasets confirmed the advantages of XCCNet. Although deep learning has shown significant potential in pneumonia detection, it still faces several challenges, including issues related to data, model generalization, computational resources, interpretability, and clinical validation, as discussed by Ren et al. (2024); Pramanik et al. (2022); Debbagh et al. (2023). In X-ray images, pneumonia lesions may exhibit low contrast with surrounding normal tissue or be affected by noise, making it difficult for the model to distinguish the pathological regions accurately. Furthermore, imaging data from different devices and hospitals may vary (e.g., image resolution, quality, acquisition angle, etc.), undermining the model’s generalization capability across different environments. For medical image analysis, clinicians must understand how the model arrives at its diagnosis. However, the limited interpretability of existing models may reduce the trust healthcare professionals place in them, affecting their clinical applicability.

To address these issues, this paper proposes an optimized detection model based on the FAST-YOLO network, exploring its application in pneumonia diagnosis using chest X-ray images. By improving the network structure of YOLOv11 and refining the training strategy, the model effectively preserves feature expression capabilities while significantly reducing computational complexity. Enhance lesions’ recognition accuracy and localization precision in pneumonia X-ray images. The Fast-YOLO network utilizes the C3k2-DCNV2-DynamicConv module, effectively maintaining feature representation while reducing computational complexity. When dealing with geometric deformations, multi-scale, and dynamic variations in scenes, the dynamic adjustment of convolutional kernel weights enhances model performance, improving computational efficiency while reducing the number of parameters. Experimental results demonstrate that the proposed FAST-YOLO model based on C3k2-DCNV2-DynamicConv accurately detects and classifies pneumonia lesions in chest X-ray images, providing clinicians with an efficient and accurate auxiliary tool. This helps accelerate the diagnosis process and improve diagnostic accuracy.

Related program will be open source in the future: https://github.com/Zhaobin7/Fast-Yolo.

## 2 Pneumonia dataset and evaluation metrics

### 2.1 Pneumonia dataset

**Figure 1:**
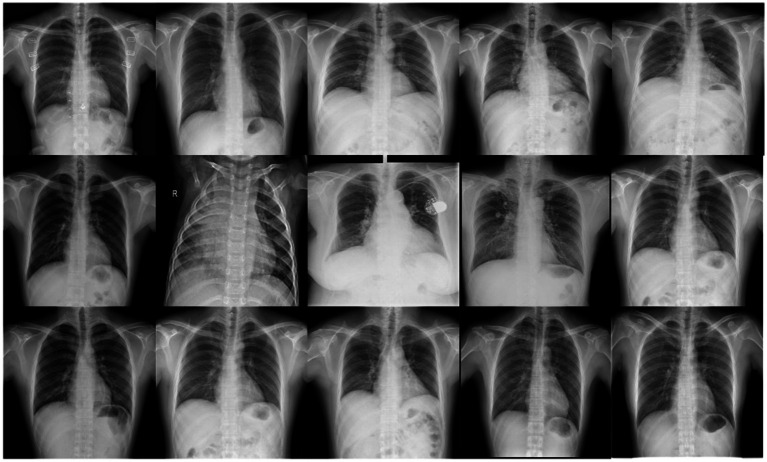
Example images from the NEU-MCD dataset. This figure shows sample chest X-ray images from the NEU-MCD pneumonia detection dataset.

MIMIC-CXR (MIMIC Chest X-ray) is an open-source chest X-ray dataset, and the dataset is designed to provide data support for medical image analysis, disease prediction, and the development of automated diagnostic systems. It contains a large number of accurately annotated chest X-ray images along with corresponding pathology reports, covering a variety of pulmonary diseases, with pneumonia being one of the key categories. The MIMIC-CXR dataset includes over 200,000 chest X-ray images, representing a diverse range of chest diseases, such as pneumonia, tuberculosis, and pneumothorax, with the majority of the images sourced from hospitalized patients. All images are stored in DICOM (Digital Imaging and Communications in Medicine) format, ensuring high resolution and clear image quality (Ali et al., 2024; Tang et al., 2024; Mabrouk et al., 2022). However, despite the detailed disease labels provided in the pathology reports, the reports are written by different radiologists, which may introduce subjectivity and label inconsistency. This variance can potentially affect the training performance of disease diagnostic models. To address this, this paper re-annotated the MIMIC-CXR dataset and constructed a YOLO-based pneumonia detection dataset, as shown in the Figure 1.

Pneumonia dataset combines field-captured images with online resources, ultimately collecting 4,194 pneumonia detection images (El-Ghandour and Obayya, 2024; Wu et al., 2024; Prince et al., 2025; Lu et al., 2025). During the experiment, the LabelImg tool was used for image annotation, completing annotation tasks for five categories: bacterial pneumonia, viral pneumonia, healthy, tuberculosis, and others. The specific annotation details are shown in Table 1.

**Table 1:** Lung condition categories and labels.

Category of labels	Number of labels
Pneumonia bacteria	1,293
Pneumonia virus	1,178
Sick	1,231
Healthy	1,188
Tuberculosis	1,366

### 2.2 Loss function and evaluation metrics

The pneumonia X-ray image detection system requires not only high-precision lesion detection but also the use of scientifically designed loss functions and evaluation metrics to optimize model performance (Liu et al., 2025; Nabeya et al., 2025). The loss function is a key element in the model training process, as its primary function is to measure the difference between the predicted results and the ground truth, providing guidance for parameter updates. A well-designed loss function can effectively balance the weights of different tasks, improving detection accuracy and robustness. Evaluation metrics play a crucial role during the testing phase, quantifying the model’s performance in real-world applications. Through these metrics, researchers and developers can systematically assess the model’s strengths and weaknesses, identifying areas for improvement and providing a basis for further optimization.

The loss function of object detection algorithms typically includes the following components:

1. Localization loss Equation 1:

(1) $$L_{loc}=\underset{i}{\sum }\lambda_{\mathrm{coord}}\left[1_{obj}\left(\begin{array}{l} {\left(x_{i}-{\hat{x}}_{i}\right)}^{2}+{\left(y_{i}-{\hat{y}}_{i}\right)}^{2}+\\ {\left(w_{i}-{\hat{w}}_{i}\right)}^{2}+{\left(h_{i}-{\hat{h}}_{i}\right)}^{2} \end{array}\right)\right]$$

Where $\lambda_{\mathrm{coord}}$ is a weight factor; $1_{obj}$ is an indicator function, when the sample contains the object, $1_{obj}$ =1. The four parameters of the bounding box are: $x_{i}$ and $y_{i}$ representing the coordinates of the center point, $w_{i}$ representing the width, and $h_{i}$ representing the height.

1. Confidence loss Equation 2:

(2) $$L_{\mathrm{conf}}=\underset{i}{\sum }1_{obj}{\left(C_{i}-{\hat{C}}_{i}\right)}^{2}+\lambda_{\mathrm{noobj}}1_{\mathrm{noobj}}{\left(C_{i}-{\hat{C}}_{i}\right)}^{2}$$

Where $C_{i}$ is the confidence of the predicted box and ${\hat{C}}_{i}$ is the confidence of the ground truth box. $1_{\mathrm{noobj}}$ is an indicator function, when the sample contains the object, $1_{\mathrm{noobj}}$ =1.

1. Classification loss Equation 3:

(3) $$L_{cls}=\underset{i}{\sum }1_{obj}\underset{c}{\sum }{\left(P_{i,c}-{\hat{P}}_{i,c}\right)}^{2}$$

Where $P_{i,c}$ is the predicted probability for the i -th box belonging to class c , and ${\hat{P}}_{i,c}$ is the actual probability of the class.

The loss function for object detection algorithms typically includes the following components:

1. Precision Equation 4:

(4) $$\mathrm{Precision}=TP/\left(TP+FP\right)\times 100\%$$

TP (True Positive) refers to the number of actual positive samples correctly predicted by the model, FP (False Positive) refers to the number of actual negative samples incorrectly predicted as positive.

1. Recall Equation 5:

(5) $$\mathrm{Recall}=TP/\left(TP+FN\right)\times 100\%$$

FN (False Negative) indicates the number of actual positive samples incorrectly predicted as negative by the model.

1. F1 score Equation 6:

(6) $$F1\;\mathrm{score}=\frac{2\times \mathrm{Precision}\times \mathrm{Recall}\;}{\;\mathrm{Precision}+\mathrm{Recall}\;}$$

1. mAP Equation 7:

(7) $$\mathrm{mAP}=\frac{-\sum_{i=1}^{n}\int_{0}^{1}P_{i}\left(R\right)dR}{N}$$

N represents the total number of sample categories, while $P_{i}\left(R\right)$ denotes the precision at a specific recall rate (Recall) for the i -th class. $AP_{i}$ (Average Precision) represents the average precision for the i th class, which is used to evaluate the detection performance of that class.

1. FPS Equation 8:

(8) PS=FigureNumber/TotalTime

FigureNumber indicates the total number of processed images, which is a key parameter in the evaluation process. TotalTime refers to the time required to process all images.

## 3 FAST-YOLO network

### 3.1 Overview of YOLOv11

YOLOv11 represents the latest advancement in the YOLO series, maintaining the efficient single-stage object detection framework typical of YOLO architectures. The architecture comprises three primary components: the Backbone, the Neck, and the Head. The Backbone is responsible for extracting multi-scale feature maps from the input images, utilizing efficient convolutional modules that enhance feature extraction capabilities and computational efficiency. These extracted feature maps are then fed into the Neck component, which further processes and fuses the features, enhancing inter-scale relationships to better capture image details and multi-scale targets. This fusion is accomplished through convolutional layers and attention mechanisms within the Neck. The Head subsequently utilizes these refined feature maps from the Neck to perform object localization and classification tasks, ultimately generating bounding boxes and class labels.

Despite YOLOv11’s strong performance in general domains, it exhibits certain limitations when processing complex medical images characterized by low contrast, high noise, or small lesions. Specifically, when images contain complicated backgrounds or small lesions with minimal contrast against the background, conventional convolutional neural networks and standard YOLO frameworks lack sufficient sensitivity and precision. To address these challenges, we integrated the C3k2-DCNv2-DynamicConv module into the YOLOv11 architecture. The C3k2 module enhances overall feature representation capabilities through efficient feature extraction. DCNv2 reduces computational complexity, improving the model’s efficiency in handling multi-scale targets, making it particularly suitable for environments with limited computational resources. DynamicConv adapts convolutional kernels dynamically based on the input image’s varying characteristics, selecting the most appropriate kernels and thus enhancing the model’s adaptability under conditions of low contrast and complex backgrounds. These enhancements significantly improve the model’s performance in complex imaging scenarios while ensuring Fast-YOLO achieves a superior balance between computational efficiency and detection accuracy.

### 3.2 C3k2-DCNV2-DynamicConv

#### 3.2.1 C3k2 module

*Feature Extraction*: The C3k2 module captures detailed features like edges, corners, and textures. The number of kernels (k) determines the feature dimensionality, with more kernels enabling more complex feature learning.

*Spatial Dimension Reduction*: A stride of 2 halves the feature map’s width and height, preserving key features while reducing computational complexity, eliminating the need for pooling, and performing downsampling via convolution.

*Computational Efficiency Optimization*: A 3×3 convolution kernel has a lower computational cost than larger kernels (such as 5×5 or 7×7 ), while still providing sufficient feature extraction capacity. Combined with stride 2, this module design is suitable for building efficient deep networks.

*Hierarchical Feature Representation*: By stacking consecutive C3k2 modules, spatial dimensional compression occurs while simultaneously increasing feature complexity along the channel dimension $\left(k\right)$ , enabling the representation of multi-level features.

#### 3.2.2 DCNV2 module

*Feature Extraction*: DCNV2 is a variant of depthwise convolution, where each input channel undergoes independent convolution, typically with 3×3 or 5×5 kernels. It also incorporates pointwise convolution, using 1×1 kernels to map each output channel of the depthwise convolution to the target channels.

*Innovation*: The key innovation of DCNV2 lies in reducing the computational load of convolution operations, making it more efficient, particularly in resource-constrained environments such as mobile or embedded devices.

*Computational Complexity Analysis*: Compared to standard convolution, DCNV2 significantly reduces computational complexity. For a standard K×K convolution with $C_{\mathrm{in}}$ input channels and $C_{\mathrm{out}}$ output channels, the computational complexity is Equation 9:


(9) $$\text{Complexity}=C_{\mathrm{in}}\times C_{\mathrm{out}}\times K^{2}\times H\times W$$

For DCNV2, the computational complexity of depthwise convolution is Equation 10:

(10) $$\text{Depthwise Convolution Complexity}=C_{\mathrm{in}}\times K^{2}\times H\times W$$

The complexity of pointwise convolution is Equation 11:

(11) $$\text{Pointwise Convolution Complexity}=C_{\mathrm{in}}\times C_{\mathrm{out}}\times H\times W$$

Thus, the total computational complexity of DCNV2 is Equation 12:


(12) $$\text{DCNV2 Complexity}=C_{\mathrm{in}}\times K^{2}\times H\times W+C_{\mathrm{in}}\times C_{\mathrm{out}}\times H\times W$$

DCNV2 is more efficient, especially when $C_{\mathrm{out}}$ is large, as pointwise convolution dominates the computation, while depthwise convolution significantly reduces unnecessary calculations.

#### 3.2.3 DynamicConv module

*Dynamic Kernel Generation*: DynamicConv generates convolution kernels based on input features, using computed representations to create kernel weights via a neural network. These kernels are dynamic and computed for each input sample based on its features.

*Adaptive Convolution Kernel Selection*: DynamicConv can also adaptively choose from a set of pre-defined kernel candidates, selecting the most suitable one based on input features.

*Efficient Feature Extraction*: By adjusting kernels to match data characteristics, DynamicConv better captures local features, especially when data varies significantly (such as different styles in image classification or varying backgrounds in object detection), improving adaptability.

### 3.3 FAST-YOLO network

**Figure 2:**
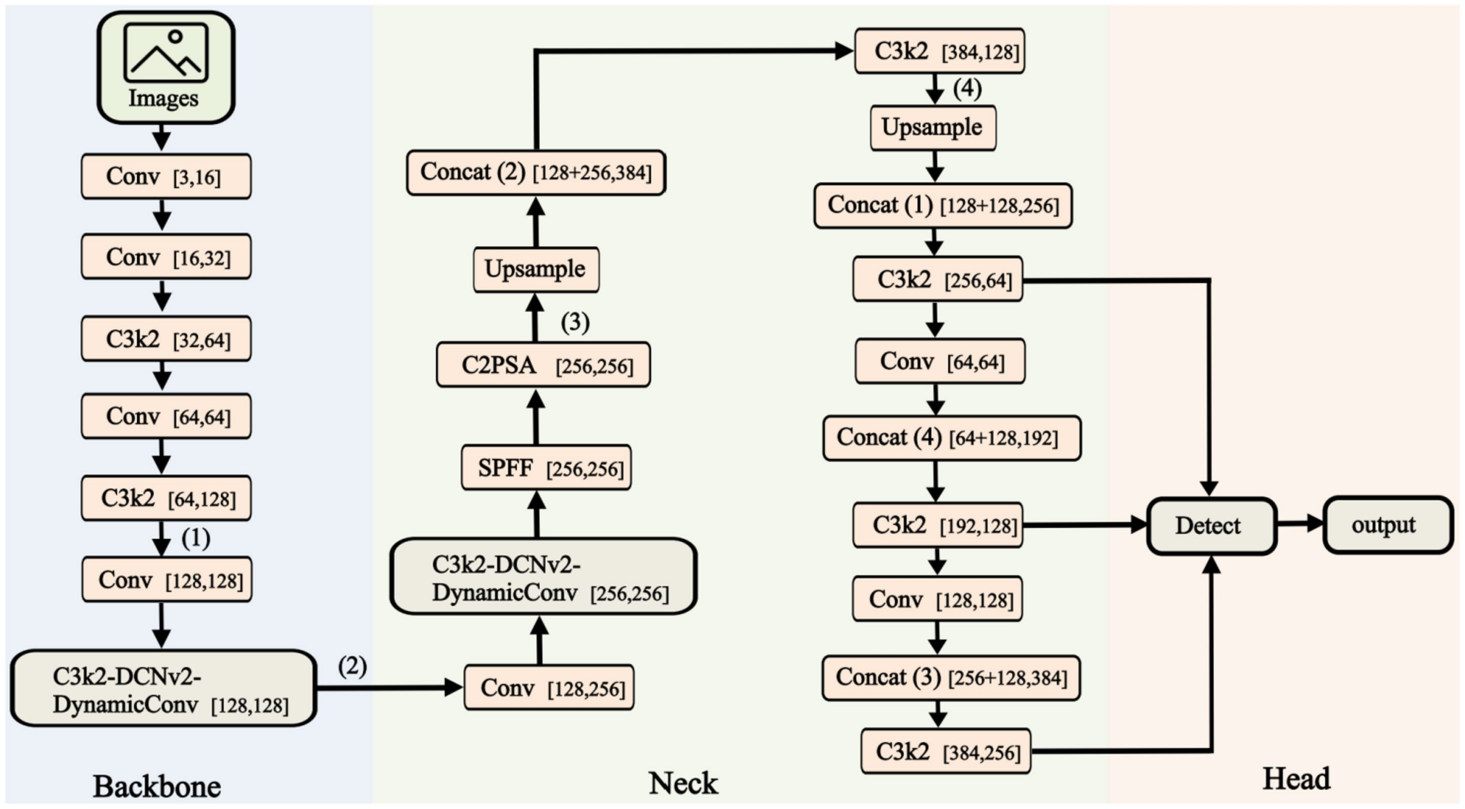
Structure of the FAST-YOLO. This figure illustrates the architecture of the Fast-YOLO network, which includes the Backbone, Neck, and Head components. The Backbone extracts features from the input images, the Neck processes and fuses these features, and the Head outputs the final detection results.

The core of YOLOv11 continues the approach of single-stage detection as seen in previous YOLO versions (Liu et al., 2025; Nabeya et al., 2025; Hakim et al., 2025; Han et al., 2025; Lekshmy and Rahiman, 2025). As shown in Figure 2, the Fast-YOLO network architecture consists of three main components: Backbone and Head for outputting target results.

1. *Backbone*: The Backbone integrates and processes extracted features to enhance semantic information. It consists of Conv, C2PSA, DynamicConv, DCNv2, and C3k2 modules. The Conv module includes convolutional layers, batch normalization (BN), and activation functions. The C2PSA module enhances fine-grained features across channels and spatial dimensions to improve detail perception. The C3k2 model maintains feature representation while reducing complexity. DCNv2 expands the receptive field and enhances feature extraction in dynamic scenarios. DynamicConv dynamically adjusts kernel weights, improving performance while reducing parameters and increasing efficiency. The Neck module boosts small-object detection by enhancing feature expression and receptive field, improving detection performance.
2. *Head*: The Head performs final regression predictions, using the Backbone’s feature map to detect bounding boxes and categories. The Upsample module restores the image’s spatial resolution to its original dimensions. The Head network uses the Generalized Intersection over Union (GIoU) loss and Weighted Non-Maximum Suppression (NMS) to optimize bounding box localization and category prediction accuracy. It adapts to targets of various sizes in complex scenes.
3. *Loss Function*: The IoU loss includes CIoU, DIoU, or EIoU to improve overlap between predicted and ground truth boxes. Classification optimization uses Label Smoothing to reduce overfitting.
4. *Data Augmentation*: Combining Mixup, Mosaic, and Copy-Paste augmentation methods enhances the model’s adaptability to complex scenes. Techniques such as CutMix and Random Erasing are used to simulate target detection in occlusion environments.

## 4 Results

The operating environment of the experimental server is shown:

**Table:** 

Name	Version.
OS Ubuntu MATE	18.04.
CPU Intel(R) Xeon(R) CPU	E5-2620 v4 @ 2.10GHz.
RAM	256GB.
GPU GeForce	RTX 3090*2.
Driver	455.23.05.
CUDA	11.1.
Python	3.7.13.
Torch	1.10.1 + cu11.
Torch vision	0.11.2++cu111.

In this paper, we disclose the hyperparameter configuration used in our experiments to ensure the reproducibility of our research. These hyperparameters were optimized experimentally to balance accuracy, efficiency, and training stability, as shown in Table 2.

**Table 2:** FAST-YOLO experiment hyperparameter configuration.

Parameter name	Parameter value
Epochs	500
Patience	100
Batch	16
Imgsz	640
Workers	8
Optimizer	auto
Seed	0
Close_mosaic	10
Amp	TRUE
Fraction	1
Mask_ratio	4
Dropout	0
Max_det	300
Lr0	0.01
Lrf	0.01
Momentum	0.937
Weight_decay	0.0005
Warmup_epochs	3
Warmup_momentum	0.8
box	7.5
Cls	0.5
Hsv_h	0.015
Hsv_s	0.7
Hsv_v	0.4
Shear	0
Perspective	0
Flipud	0
Fliplr	0.5
Bgr	0
Mosaic	1
Mixup	0
Translate	0.1
Scale	0.5
Degrees	0
Copy_paste	0

### 4.1 Pneumonia X-ray image detection

**Figure 3:**
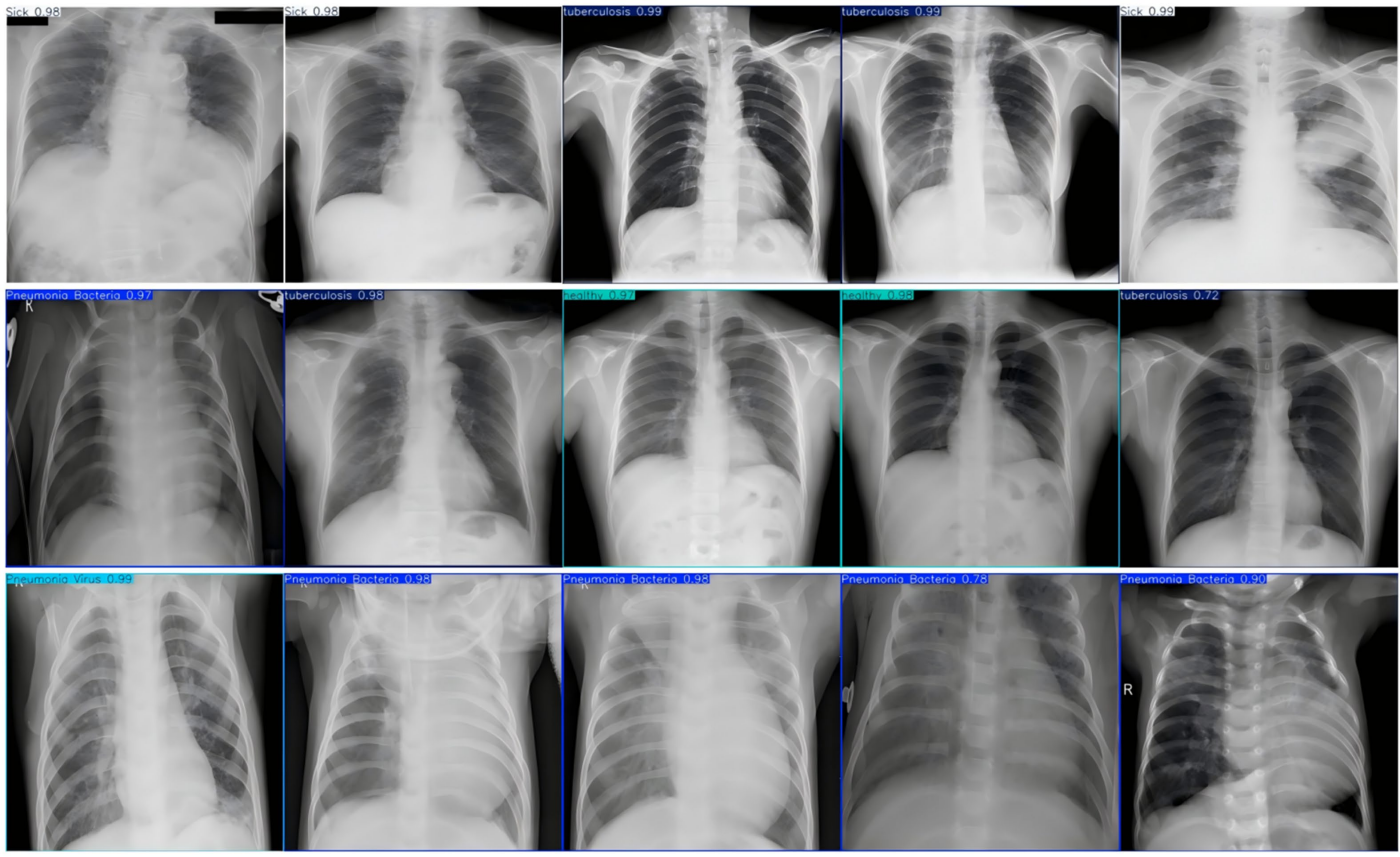
X-ray image test for pneumonia. This figure presents chest X-ray images from the Fast-YOLO model, with detected conditions labeled along with their associated confidence scores. These scores represent the model’s confidence in the presence of the detected conditions, ranging from 0 to 1.

To evaluate the performance of the FAST-YOLO algorithm in multi-object detection tasks, this study conducted comparative experiments between FAST-YOLO and other mainstream algorithms. A unified dataset and configuration parameters are used throughout the experimental process. The experimental results are shown in Figure 3, where the values annotated within the recognition boxes represent confidence scores. This metric is a crucial indicator for assessing the reliability of the algorithm’s object detection in images. The confidence score can be regarded as a model’s assessment of the probability of the presence of a particular object, with values ranging from 0 to 1. Higher confidence values indicate a higher level of certainty in the model’s judgment regarding the object’s presence.

**Figure 4:**
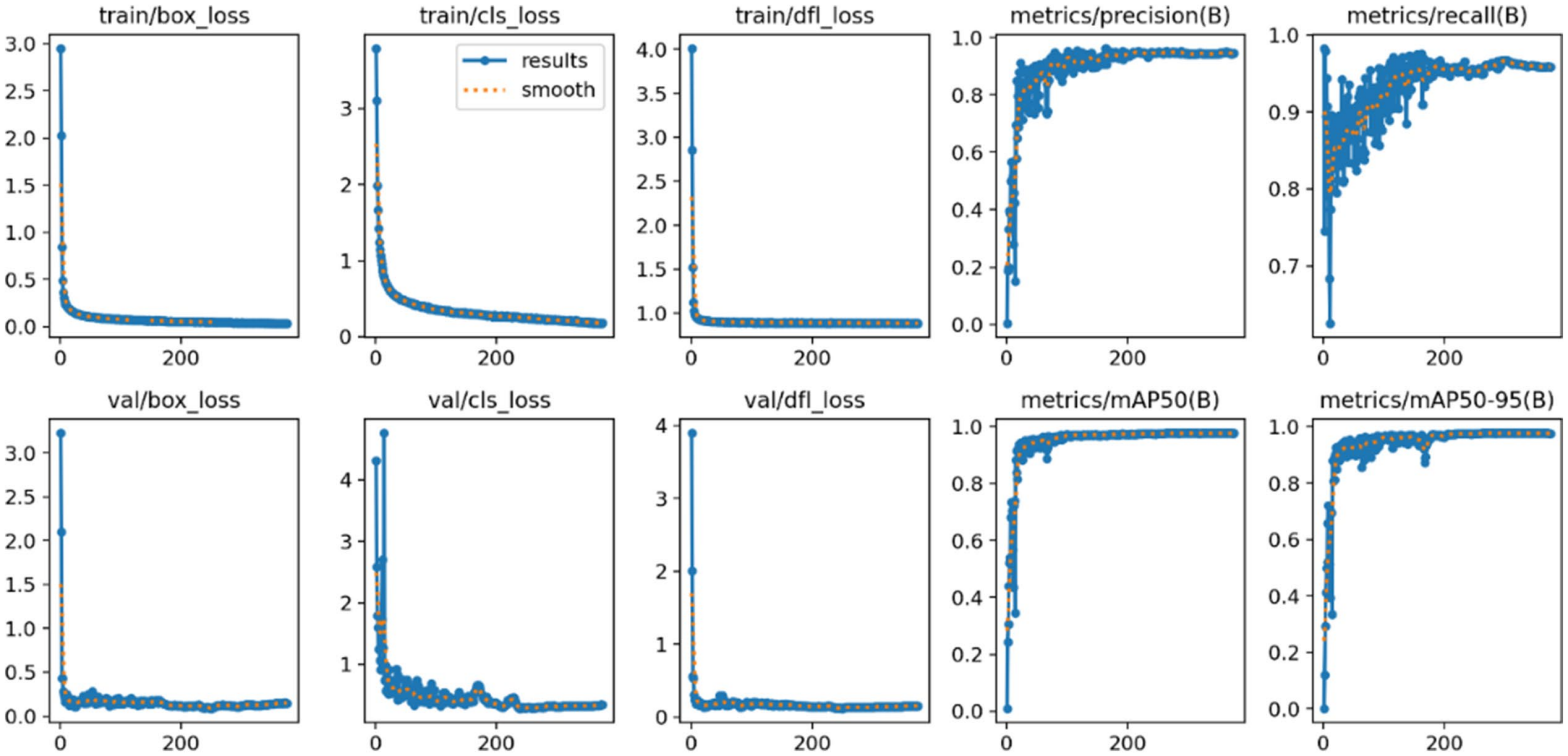
FAST-YOLO algorithm training process. This figure shows the training process of the Fast-YOLO algorithm over 500 epochs. The model begins to converge after approximately 80 epochs, with performance metrics such as precision, recall, and mAP@0.5 approaching high values.

As illustrated in Figure 4, during the training process spanning 500 epochs, the FAST-YOLO model approaches convergence at approximately the 80th epoch. Moreover, the precision, recall, and mAP@0.5 values and accuracy are all close to 100%. This demonstrates that the FAST-YOLO model, due to the incorporation of C3k2-DCNV2-DynamicConv, exhibits superior performance in terms of convergence speed as well as precision, recall, mAP@0.5, and mAP@0.5:0.95.

**Figure 5:**
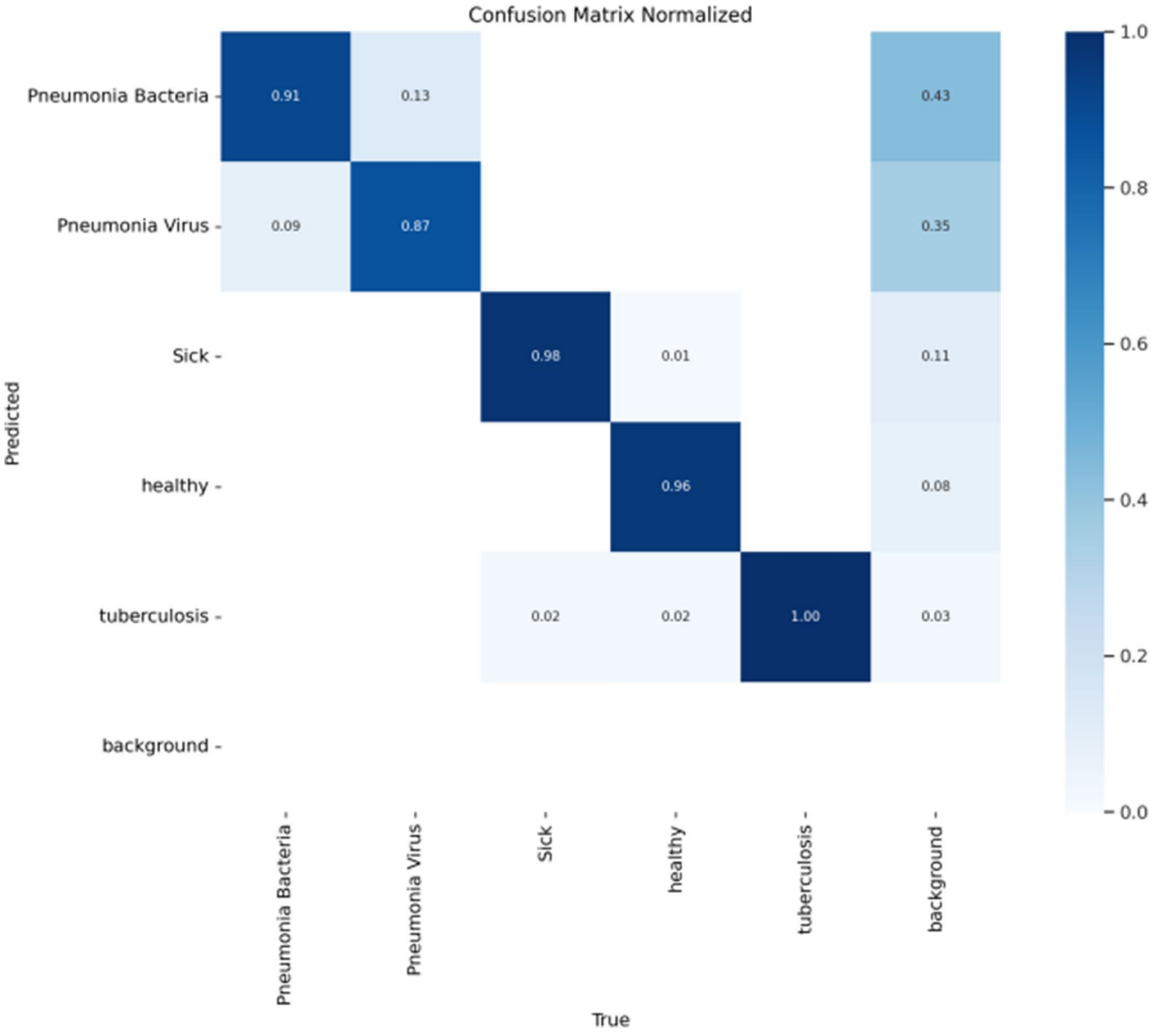
Confusion matrix evaluates the performance of classification models. This figure presents the confusion matrix used to assess the classification performance of the Fast-YOLO model. It shows the model’s ability to correctly classify pneumonia and healthy images during the detection process.

In object detection tasks, each detection result typically requires the assignment of a class label to evaluate the model’s classification performance across different categories. After training the FAST-YOLO model, a confusion matrix is generated using the test set to comprehensively assess the model’s overall performance. As shown in Figure 5, the results indicate that the FAST-YOLO’s classification performance is satisfactory, providing a more thorough evaluation of the model’s actual performance in object detection tasks, thereby offering strong support for subsequent optimization and improvements.

**Figure 6:**
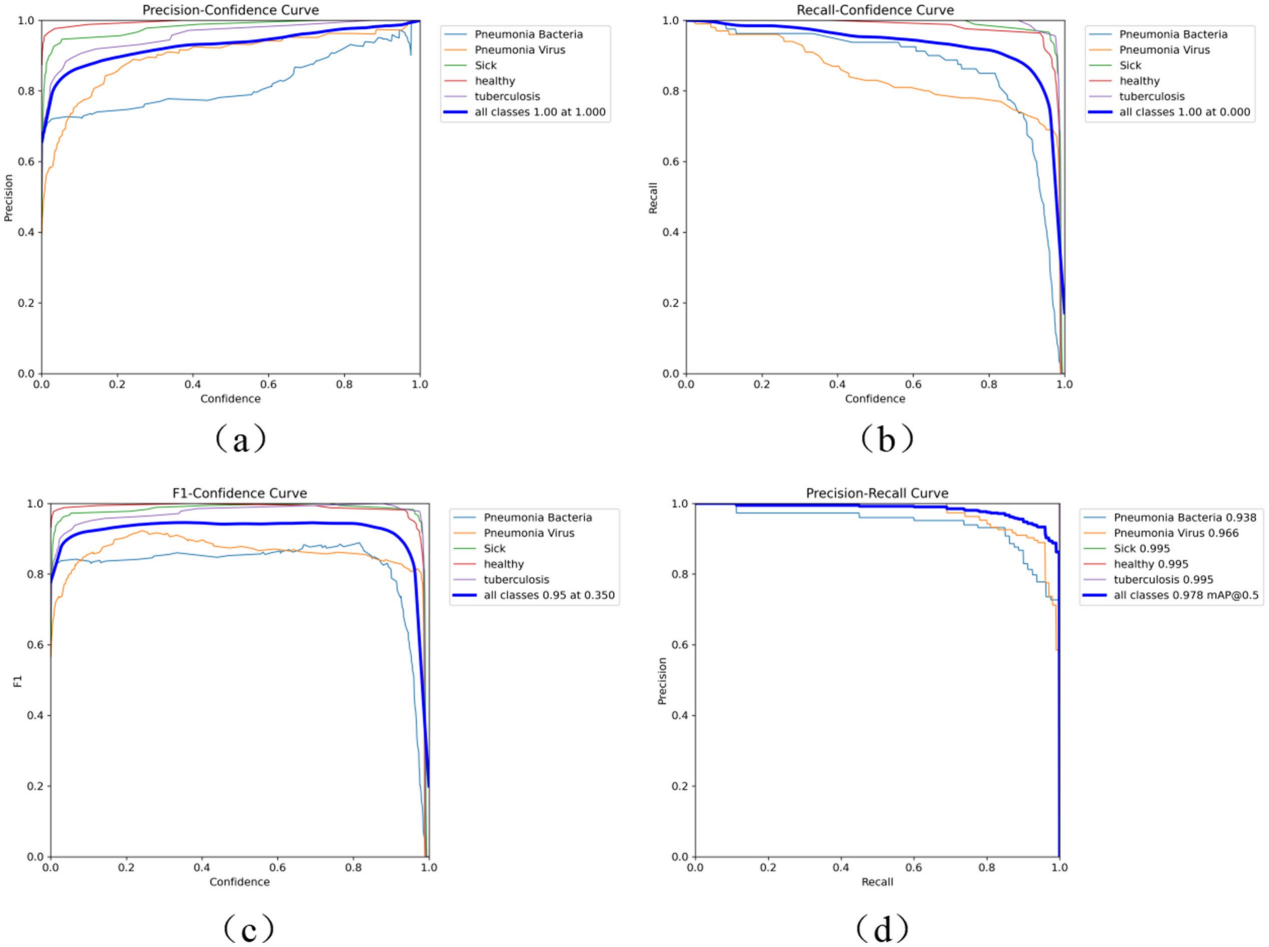
**(a)** P curve, **(b)** R curve, **(c)** F1 curve, and **(d)** PR curve performance indicators. This figure displays performance curves for the Fast-YOLO model, including the Precision Curve (P curve), Recall Curve (R curve), F1 Curve, and Precision-Recall (PR) Curve. These curves help assess the model’s performance in different task scenarios.

As shown in Figure 6, the performance of the Fast-YOLO model is comprehensively evaluated using performance metrics such as the P-curve, R-curve, F1-curve, and PR curve. These metrics provide multidimensional perspectives for analyzing the model’s strengths and weaknesses in different task scenarios, effectively revealing its overall performance characteristics. The P-curve (Precision Curve) primarily reflects the model’s false positive rate, evaluating its performance in reducing erroneous detections by displaying precision variations at different thresholds. The R-curve (Recall Curve) reveals the model’s false negative rate, showing its ability to identify true targets in object detection tasks. The F1 curve, based on the weighted harmonic mean of precision and recall, assesses the balance between accuracy and completeness in the model’s performance. The PR curve (Precision-Recall Curve) further demonstrates the trade-off between precision and recall at different thresholds, offering a more comprehensive performance evaluation, especially when addressing class imbalance issues. By analyzing these four metrics, the overall performance of the Fast-YOLO model in multiple key dimensions can be assessed. The results show that Fast-YOLO exhibits outstanding performance across various evaluation indicators, confirming its practical application value in complex task environments.

### 4.2 Comparison of performance across different detection models

In order to validate the effectiveness of the proposed method for pneumonia X-ray image detection and assess the performance of the improved algorithm, a comparison is conducted under the same dataset and experimental conditions. The networks are trained for 500 epochs, and the improved model is tested alongside lightweight object detection models, including YOLOv7-Tiny, YOLOv5s, YOLOv5n, YOLOv3-Tiny, D-FINE-L, RTMDet-L, and YOLOv3-spp (Liu et al., 2025; Nabeya et al., 2025; Hakim et al., 2025; Han et al., 2025; Lekshmy and Rahiman, 2025; Lyu et al., 2022; Peng et al., 2024). The test results are shown in Table 3. Fast-YOLO demonstrates exceptional performance across multiple evaluation metrics, effectively validating its superiority in X-ray pneumonia diagnosis. Fast-YOLO significantly outperforms other algorithms with a frames per second (FPS) rate of 150. A higher FPS indicates that Fast-YOLO can rapidly process images, crucial for real-time diagnosis. The precision of Fast-YOLO reaches 95.2%, surpassing other models, indicating higher accuracy in identifying pneumonia lesions and reducing false detection rates, thereby enhancing the reliability of diagnostic results. Fast-YOLO also achieves a recall of 94.9%, the highest among all algorithms. It demonstrates its greater sensitivity in detecting pneumonia-affected regions and effectively minimizing missed diagnoses, which is significant for critical lesions in medical diagnostics. Furthermore, in the comprehensive metric of mAP@0.5:0.95, Fast-YOLO excels with a score of 97.8%, significantly outperforming other algorithms. This metric evaluates the algorithm’s performance across various intersection-over-union (IOU) thresholds, highlighting Fast-YOLO’s stability and robustness in different detection tasks. Overall, Fast-YOLO excels in precision, recall, and processing speed and outperforms other YOLO versions across multiple metrics, confirming its considerable advantages in the application of X-ray pneumonia diagnosis.

**Table 3:** The performance comparison of the different algorithms.

Algorithms	FPS	precision	recall	mAP@0.5	mAP@0.5:0.95
YOLOv5n	103	94.6%	94.2%	97%	77.7%
YOLOv5s	90	95%	96.3%	98.3%	83%
YOLOv7-Tiny	91	93.9%	96.2%	97.9%	79.8%
YOLOv11	55	94.4%	95.8%	97.6%	97.6%
Fast-YOLO	120	95.2%	94.9%	97.8%	97.8%
RTMDet-L	46	94.2%	95.1%	97.3%	82.5%
D-FINE-L	51	94.8%	95.5%	97.7%	85.9%

### 4.3 Generalization experiment of fast-YOLO

The previous experiments have demonstrated the excellent performance of the Fast-YOLO algorithm on multi-object work piece classification datasets. To investigate the generalization capabilities of the Fast-YOLO network further and analyze its detection performance on other publicly available datasets, this section collects the open-source dataset for welding parts defect detection and fish classify detection for experimentation.

**Figure 7:**
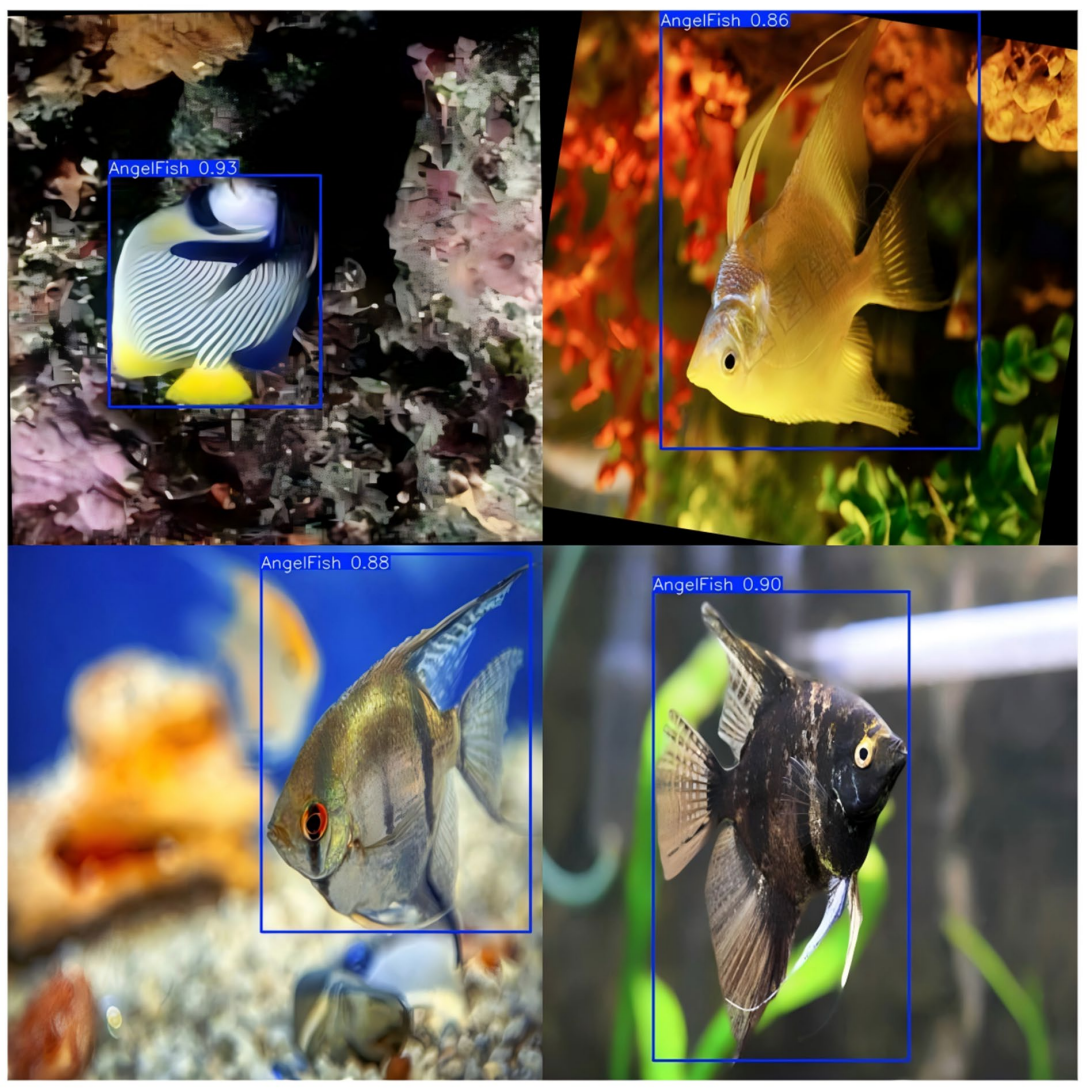
Experiment of fish classification detection. This figure shows the results of the fish classification detection experiment using the Fast-YOLO model. The model detects multiple fish species, with detection boxes around each fish and their corresponding classification scores.

The experimental results of fish detection are shown in Figure 7. There are 13 categories of fish detection: angel fish, blue tang, butterfly fish, clown fish, goldfish, gourami, morish idol, platy fish, ribboned sweetlips, three striped damsel fish, yellow cichlid, yellow tang, and zebrafish.

As shown in Table 4, Fast-YOLO demonstrates superior performance in fish detection research, with a significant advantage in FPS, enabling faster completion of fish detection tasks, particularly in real-time processing applications. The high recall rate of Fast-YOLO indicates its ability to effectively detect a more significant number of fish targets, reducing missed detections and ensuring the comprehensiveness of the results. Fast-YOLO also excels in mAP@0.5 and mAP@0.5:0.95, demonstrating a decisive advantage in detection accuracy and object localization capabilities. Its detection performance is more stable across different IoU thresholds, meeting the demands of various detection conditions. The model’s robust real-time processing ability and high-precision detection results enhance its applicability in dynamic environments.

**Table 4:** The performance comparison of the different algorithms.

Algorithms	FPS	precision	recall	mAP@0.5	mAP@0.5:0.95
YOLOv5n	101	64.7%	75.3%	74.6%	36.0%
YOLOv5s	99	57.6%	68.4%	69.6%	31.0%
YOLOv7-Tiny	94	61.5%	69.1%	73.0%	36.0%
YOLOv11	76	89.4%	89.5%	93.3%	79.7%
Fast-YOLOv11	108	90.6%	89.4%	94.3%	73.6%

**Figure 8:**
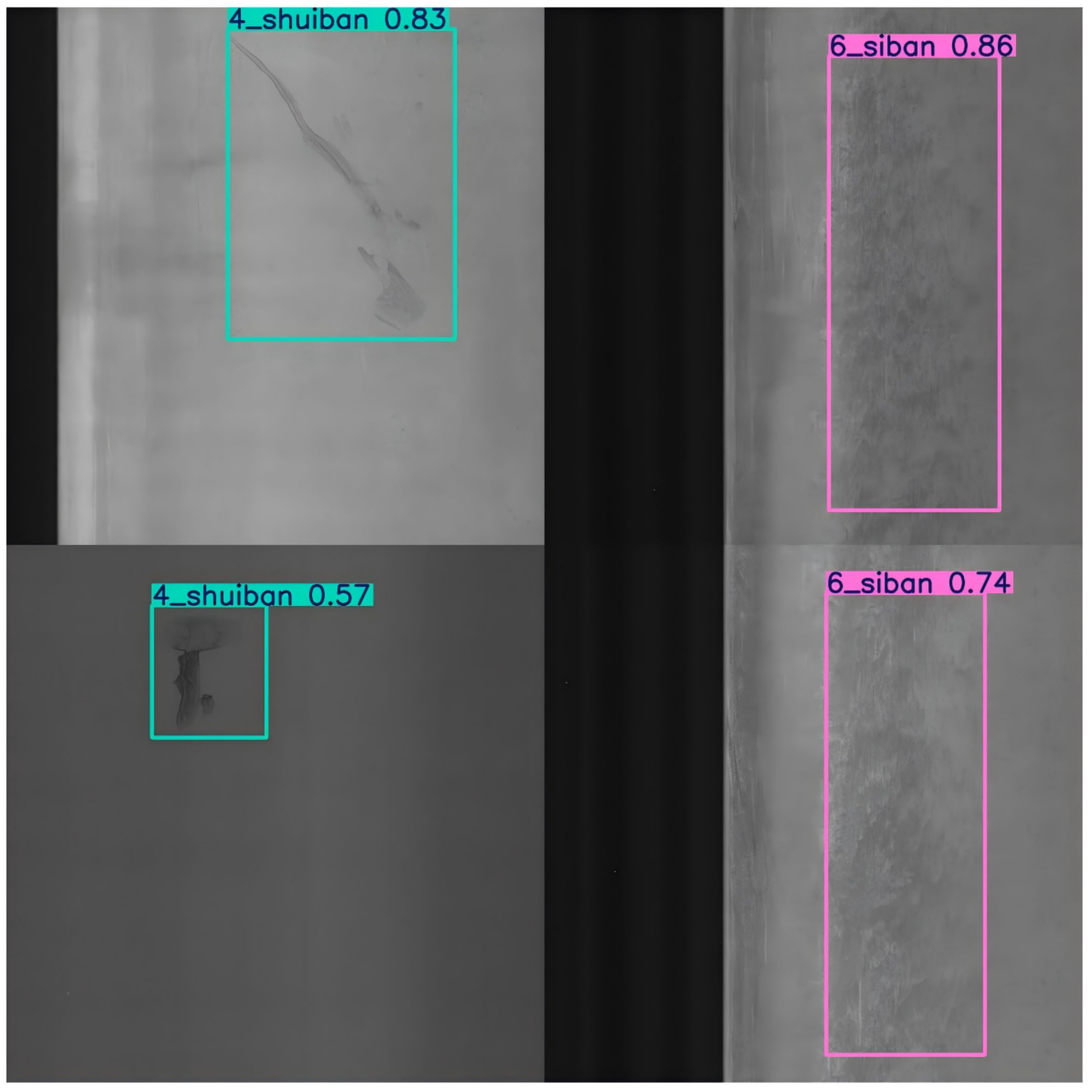
Experiment of welding parts defect detection. This figure shows the results of the welding parts defect detection experiment using the Fast-YOLO model. The model detects various defects in welded components, with detection boxes around the identified defects and their corresponding confidence scores.

The experimental results of welded defect detection are shown in Figure 7. The welded surface defect detection components are classified into eleven categories: perforation, weld seam, crescent bend, water stain, oil stain, four plates, foreign object, indentation, shock mark, flexural fracture, and scratch (see Figure 8).

As shown in Table 5, Fast-YOLO exhibits significantly better FPS performance in detecting surface defects in welded components than other YOLO algorithms, enabling rapid feedback in industrial production and reducing detection delays. Although YOLOv7-Tiny slightly outperforms in terms of precision, the advantages of Fast-YOLO in recall and FPS speed compensate for the gap in accuracy, resulting in superior overall performance. The higher recall of Fast-YOLO indicates greater sensitivity in defect identification, allowing for more comprehensive detection of surface defects in welded components and minimizing missed detection. The mAP@0.5 and mAP@0.5:0.95 metrics, which assess the algorithm’s performance across multiple IOU thresholds, demonstrate that Fast-YOLO’s detection capability is more robust under various conditions, making it particularly suitable for the detection of variable surface defects in welded components.

**Table 5:** The performance comparison of the different algorithms.

Algorithms	FPS	precision	recall	mAP@0.5	mAP@0.5:0.95
YOLOv5n	107	67.4%	43.9%	49.3%	22.3%
YOLOv5s	93	64.3%	51.3%	53.6%	24.3%
YOLOv7-Tiny	94	73.3%	49.9%	54.5%	24.3%
YOLOv11	57	53%	65.6%	64.3%	33.2%
Fast-YOLOv11	112	69.7%	61.6%	65.1%	34.0%

From the above data, it can be found that the related evaluation indicators get better results in different data sets, which fully proves the generalization performance of the Fast-YOLO network.

## 5 Ablation experiment

We conducted ablation experiments by individually removing the C3k2, DCNv2, and DynamicConv modules to verify their respective effectiveness.

Removing the DCNv2 module resulted in Fast-YOLO achieving an FPS of 113, slightly lower than the original model’s FPS, along with decreases in precision, recall, mAP@0.5, and mAP@0.5:0.95. This indicates that the DCNv2 module effectively enhances the model’s feature extraction capabilities and expands its receptive field.

When removing the C3k2 module, Fast-YOLO’s FPS significantly dropped to 53. Although precision, recall, mAP@0.5, and mAP@0.5:0.95 metrics showed slight improvements, the computational efficiency was greatly reduced. This demonstrates that the C3k2 module effectively maintains feature representation while significantly reducing computational complexity.

Removing the DynamicConv module decreased Fast-YOLO’s FPS to 67, accompanied by slight reductions in various detection accuracy metrics. This shows that the DynamicConv module effectively reduces model parameters and enhances computational efficiency.

Overall, the experimental results indicate that integrating C3k2, DCNv2, and DynamicConv modules allows Fast-YOLO to achieve an optimal balance between detection accuracy and computational efficiency, confirming the effectiveness of the combined modules (see Table 6).

**Table 6:** Ablation study on the performance impact of different components.

Algorithms	FPS	precision	recall	mAP@0.5	mAP@0.5:0.95
YOLOv11	55	94.4%	95.8%	97.6%	97.6%
Fast-YOLO	120	95.2%	94.9%	97.8%	97.8%
Fast-YOLO without C3k2	53	95.9%	95.4%	97.9%	97.8%
Fast-YOLO without DCNV2	113	93.9%	94.7%	97.1%	96.7%
Fast-YOLO without DynamicConv	67	95.3%	95.7%	97.5%	97.4%

## 6 Broader impact

In clinical settings, the quality of X-ray images is often affected by factors such as equipment variability, imaging angles, and noise, which may result in low contrast or distortion. To address the diversity in sources and quality of clinical images, the Fast-YOLO model employs data augmentation techniques to diversify training data. Moreover, the network structure of Fast-YOLO has been specifically optimized to maintain robust feature extraction capabilities, particularly for images affected by low contrast or noise. By integrating the C3k2 module, DCNv2, and DynamicConv, the model efficiently and accurately identifies pneumonia lesions even under poor image quality conditions.

In addition, Fast-YOLO exhibits excellent computational efficiency, enabling real-time processing of large volumes of X-ray images in high-workload clinical environments. During emergencies, this rapid image-processing capability assists physicians in making timely diagnostic decisions, thus significantly improving clinical workflow efficiency and diagnostic speed. These advantages facilitate the effective integration of Fast-YOLO into routine hospital workflows.

## 7 Conclusion

To address the limitations of traditional YOLO models in detecting small targets and recognizing low-contrast lesions in pneumonia X-ray images, structural optimizations and parameter adjustments were made to the YOLOv11 model. The proposed pneumonia diagnosis method based on the Fast-YOLO deep learning model integrates image enhancement techniques with network structure optimization, significantly improving the efficiency and accuracy of pneumonia detection. By redesigning the YOLOv11 network and incorporating the C3k2 model, DCNv2 module, and DynamicConv mechanism, the model effectively tackles challenges such as low contrast, uneven distribution of local lesions, and geometric deformations in pneumonia X-ray images, thereby enhancing feature extraction capability and computational efficiency in complex environments. Experimental results demonstrate that, compared to other mainstream object detection models, the Fast-YOLO model outperforms traditional convolutional neural network methods in comprehensive performance. Additionally, it offers significant computational resource advantages. In comparison to other widely used detection models, Fast-YOLO not only improves detection speed but also excels in generalization, meeting the diagnostic needs for pneumonia images across various real-world scenarios, highlighting its significant application value and potential for widespread adoption.

Despite the promising performance of the Fast-YOLO optimization model in pneumonia X-ray image detection, certain limitations still exist. While the model generally shows high detection accuracy, its ability to detect tiny lesions remains an area for further improvement. Future research could explore enhancing the network architecture, incorporating multi-scale feature fusion strategies, and strengthening noise resistance to improve further the model’s stability and robustness in complex environments. The pneumonia X-ray image detection method based on the Fast-YOLO optimized model offers an efficient and accurate solution for the early diagnosis of pneumonia with promising clinical applications. As the model architecture continues to be optimized and computational hardware performance improves, Fast-YOLO is expected to demonstrate more significant application potential in object detection. Future research can integrate larger-scale clinical data and multi-modal information (such as CT images or patient history data) to refine the detection algorithms further, thereby enhancing the accuracy and applicability of automated pneumonia diagnosis systems. This contributes to the advancement of intelligent healthcare and provides crucial support for auxiliary diagnosis in medical practice.

## Data Availability

The original contributions presented in the study are included in the article/supplementary material, further inquiries can be directed to the corresponding author.
